# Supplementary material for: Exponential Random Graph Modeling for Complex Brain Networks
Source: PLoS One. 2011 May 25;6(5):e20039. doi: 10.1371/journal.pone.0020039 (PMC3102079; doi:10.1371/journal.pone.0020039)
Supplement: Appendix S1 — Model Selection (PDF) [file pone.0020039.s001.pdf]

Appendix S1: Model Selection

The following model/metric selection methods are based on different criteria (p-value, AIC, graphical GOF) but follow the same steps for comparability. Steps 1 and 2 aim to select the best Connectedness and Local Efficiency metrics respectively from the two in **Table 2**. We opted to select one of each to avoid potential collinearity issues that may arise given that metrics within the same category attempt to capture the same properties. We started with selection of the Connectedness metric first as inclusion of both Edges and Two-Path in the model generally led to non-convergence. The location metric Nodematch was assessed last as we wanted to examine whether we could capture nodal information after accounting for topological structure. This set of steps merely provides a needed ad hoc procedure for metric selection in this context and is only one of many possible approaches. Fitting all possible models is generally infeasible (especially as more explanatory metrics are considered); thus, parsimonious approaches like the one outlined here are needed.

<u>*P-Value Approach*</u>:

<u>Step 1</u> – Selection of Connectedness metric.

Fit $P_{\boldsymbol{\theta}}(\boldsymbol{Y}=\boldsymbol{y})=\kappa(\boldsymbol{\theta})^{-1}\exp\{\theta_1\text{Edges}+\theta_2\text{GWESP}+\theta_3\text{GWDSP}+\theta_4\text{GWNSP}+\theta_5\text{GWD}\}$ and $P_{\boldsymbol{\theta}}(\boldsymbol{Y}=\boldsymbol{y})=\kappa(\boldsymbol{\theta})^{-1}\exp\{\theta_1\text{Two-Path}+\theta_2\text{GWESP}+\theta_3\text{GWDSP}+\theta_4\text{GWNSP}+\theta_5\text{GWD}\}$.

Retain Connectedness metric (Edges or Two-Path) with smallest p-value, which we will denote as C.

<u>Step 2</u> – Selection of Local Efficiency metric.

Fit $P_{\boldsymbol{\theta}}(\boldsymbol{Y}=\boldsymbol{y})=\kappa(\boldsymbol{\theta})^{-1}\exp\{\theta_1\text{C}+\theta_2\text{GWESP}+\theta_3\text{GWNSP}+\theta_4\text{GWD}\}$ and $P_{\boldsymbol{\theta}}(\boldsymbol{Y}=\boldsymbol{y})=\kappa(\boldsymbol{\theta})^{-1}\exp\{\theta_1\text{C}+\theta_2\text{GWDSP}+\theta_3\text{GWNSP}+\theta_4\text{GWD}\}$. Retain Local Efficiency metric (GWESP or GWDSP) with smallest p-value, which we will denote as LE.

<u>Step 3</u> – Backward Selection.

Implement a traditional backward selection approach (with $\alpha = 0.1$) to reduce the model $P_{\boldsymbol{\theta}}(\boldsymbol{Y}=\boldsymbol{y})=\kappa(\boldsymbol{\theta})^{-1}\exp\{\theta_1\text{C}+\theta_2\text{LE}+\theta_3\text{GWNSP}+\theta_4\text{GWD}\}$ as appropriate. This reduced model will be denoted as $P_{\boldsymbol{\theta}}(\boldsymbol{Y}=\boldsymbol{y})=\kappa(\boldsymbol{\theta})^{-1}\exp\{\boldsymbol{\theta}_r^{\mathrm{T}}\mathbf{g}_r(\boldsymbol{y})\}$. A discussion of the backward selection approach (in the context of linear regression) can be found in [30].

<u>Step 4</u> – Selection of final model.

Fit $P_{\boldsymbol{\theta}}(\boldsymbol{Y}=\boldsymbol{y})=\kappa(\boldsymbol{\theta})^{-1}\exp\{\boldsymbol{\theta}_r^{\mathrm{T}}\mathbf{g}_r(\boldsymbol{y})+\theta_{r+1}\text{Nodematch}\}$ to determine whether adding information about nodal location in the brain leads to a "better" model. If the p-value for the estimate of $\theta_{r+1}\leq\alpha(=0.1)$ then the final model is $P_{\boldsymbol{\theta}}(\boldsymbol{Y}=\boldsymbol{y})=\kappa(\boldsymbol{\theta})^{-1}\exp\{\boldsymbol{\theta}_r^{\mathrm{T}}\mathbf{g}_r(\boldsymbol{y})+\theta_{r+1}\text{Nodematch}\}$; otherwise, the final model is $P_{\boldsymbol{\theta}}(\boldsymbol{Y}=\boldsymbol{y})=\kappa(\boldsymbol{\theta})^{-1}\exp\{\boldsymbol{\theta}_r^{\mathrm{T}}\mathbf{g}_r(\boldsymbol{y})\}$.

AIC Approach:

Steps 1 and 2

Same as in the p-value approach except now the C and LE metrics are taken from the models with the lower AIC values [16].

Step 3 – AIC Selection.

Fit $P_{\boldsymbol{\theta}}(\boldsymbol{Y}=\boldsymbol{y})=\kappa(\boldsymbol{\theta})^{-1}\exp\{\theta_1\mathrm{C}+\theta_2\mathrm{LE}+\theta_3\mathrm{GWNSP}+\theta_4\mathrm{GWD}\}$ and all $\binom{4}{3}=4$ possible models containing three explanatory metrics (since all p-value selection models ended up having at least three terms). Compare the 5 models and select the one with the lowest AIC value, which will be denoted as $P_{\boldsymbol{\theta}}(\boldsymbol{Y}=\boldsymbol{y})=\kappa(\boldsymbol{\theta})^{-1}\exp\{\boldsymbol{\theta}_r^{\mathrm{T}}\mathbf{g}_r(\boldsymbol{y})\}$.

Step 4 – Selection of final model.

Same as in the p-value approach except now the final model is the one with the lower AIC value between $P_{\boldsymbol{\theta}}(\boldsymbol{Y}=\boldsymbol{y})=\kappa(\boldsymbol{\theta})^{-1}\exp\{\boldsymbol{\theta}_r^{\mathrm{T}}\mathbf{g}_r(\boldsymbol{y})+\theta_{r+1}\mathrm{Nodematch}\}$ and $P_{\boldsymbol{\theta}}(\boldsymbol{Y}=\boldsymbol{y})=\kappa(\boldsymbol{\theta})^{-1}\exp\{\boldsymbol{\theta}_r^{\mathrm{T}}\mathbf{g}_r(\boldsymbol{y})\}$.

Graphical GOF Approach:

Steps 1 and 2

Same as in the p-value approach except now the C and LE metrics are taken from the models with the better graphical GOF plots [17]. Examples of these types of plots are exhibited in **Figures 3-13**. The vertical axis is the logit of relative frequency, the solid lines represent the statistics of the observed network, and the boxplots represent the distributions of 100 simulated networks based on the fitted ERGM. For good-fitting models, the plot of the observed network should closely match that of the simulated networks as in **Figure 8**. We can see that in this figure the model does a good job of capturing the geodesic (global efficiency), shared partner (local efficiency), degree, and triad census (motifs) distributions. For our purposes, we considered each of these four GOF plots equally in making the subjective decisions about model fits. For a further discussion of these comparison statistics see [29].

Step 3 – Graphical GOF Selection.

Same as in the AIC approach except now the model (of the 5 models) with the best GOF plots will be selected. This model is again denoted as $P_{\boldsymbol{\theta}}(\boldsymbol{Y}=\boldsymbol{y})=\kappa(\boldsymbol{\theta})^{-1}\exp\{\boldsymbol{\theta}_r^{\mathrm{T}}\mathbf{g}_r(\boldsymbol{y})\}$.

Step 4 – Selection of final model.

Same as in the AIC approach except now the final model is the one with the better GOF plots between $P_{\boldsymbol{\theta}}(\boldsymbol{Y}=\boldsymbol{y})=\kappa(\boldsymbol{\theta})^{-1}\exp\{\boldsymbol{\theta}_r^{\mathrm{T}}\mathbf{g}_r(\boldsymbol{y})+\theta_{r+1}\mathrm{Nodematch}\}$ and $P_{\boldsymbol{\theta}}(\boldsymbol{Y}=\boldsymbol{y})=\kappa(\boldsymbol{\theta})^{-1}\exp\{\boldsymbol{\theta}_r^{\mathrm{T}}\mathbf{g}_r(\boldsymbol{y})\}$.
